# *SOX18* Is a Novel Target Gene of Hedgehog Signaling in Cervical Carcinoma Cell Lines

**DOI:** 10.1371/journal.pone.0143591

**Published:** 2015-11-20

**Authors:** Isidora Petrovic, Milena Milivojevic, Jelena Popovic, Marija Schwirtlich, Branislava Rankovic, Milena Stevanovic

**Affiliations:** Laboratory for Human Molecular Genetics, Institute of Molecular Genetics and Genetic Engineering, University of Belgrade, P.O.BOX 23, 11000 Belgrade, Serbia; Indiana University School of Medicine, UNITED STATES

## Abstract

Although there is much evidence showing functional relationship between Hedgehog pathway, in particular Sonic hedgehog, and SOX transcription factors during embryonic development, scarce data are available regarding their crosstalk in cancer cells. SOX18 protein plays an important role in promoting tumor angiogenesis and therefore emerged as a promising potential target in antiangiogenic tumor therapy. Recently it became evident that expression of *SOX18* gene in tumors is not restricted to endothelium of accompanying blood and lymphatic vessels, but in tumor cells as well.In this paper we have identified human *SOX18* gene as a novel target gene of Hedgehog signaling in cervical carcinoma cell lines. We have presented data showing that expression of *SOX18* gene is regulated by GLI1 and GLI2 transcription factors, final effectors of Hedgehog signaling, and that modulation of Hedgehog signaling activity in considerably influence *SOX18* expression. We consider important that Hedgehog pathway inhibitors reduced *SOX18* expression, thus showing, for the first time, possibility for manipulationwith *SOX18* gene expression. In addition, we analyzed the role of SOX18 in malignant potential of cervical carcinoma cell line, and showed that its overexpression has no influence on cells proliferation and viability, but substantially promotes migration and invasion of cells *in vitro*. Pro-migratory effect of SOX18 suggests its role in promoting malignant spreading, possibly in response to Hedgehog activation.

## Introduction

The Hedgehog (HH) signaling pathway plays important role during normal cell differentiation and embryonic development while it is largely suppressed in the adult[1]. Pathway activation is initiated by binding of one out of three HH ligand proteins: Sonic Hedgehog (SHH); Indian Hedgehog (IHH) or Desert Hedgehog (DHH), to a transmembrane receptor protein patched (PTCH)[2,3]. In the absence of HH ligands, PTCH functions as an inhibitor of another transmembrane protein smoothened (SMO). Binding of any of the HH ligands to PTCH receptor relieves the suppression of SMO, resulting in downstream activation of final effectors, GLI transcription factors (GLI1, GLI2 and GLI3)[1,4]. By releasing the inhibition of SMO, HH-PTCH-GLI signaling cascade is transmitted to downstream target genes.

The improper regulation of HH signaling has been linked to the etiology of many cancers[5–7]. HH pathway contribution to carcinogenesis involves several mechanisms, including mutations in PTCH and SMO receptors, overexpression of HH ligands or non-canonical activation of HH target genes[8,9]. Also, recent studies revealed the role of HH signaling in chemotherapy and radiotherapy resistance. These include resistance to docetaxel, tamoxifen and radiotheraphy in prostate, breast and pancreatic cancer patients, respectively[10–13].

*SOX18* gene is a member of a large family of diverse and well-conserved genes encoding transcription factors implicated in various developmental processes[14,15]. Previously, it has been shown that SOX18, together with SOX7 and SOX17, has an important role in vascular development and postnatal neovascularization[16,17]. Murine *Sox18*gene is reexpressed in endothelial cells of the newly formed blood vasculatureunder pathological conditions such as wound healing or tumor growth, where it is involved in endothelial cell proliferation and migration, and the establishment of vascular integrity[18,19]. Recently, it become evident that the expression of *SOX18* gene in tumors is not restricted simply to the endothelium of accompanying blood and lymphatic vessels, and that its role in tumor development and progression might go beyond regulation of tumor angiogenesis and lyphangiogenesis[20].

Literature data indicate that HH signaling does not work independently during cancer development and metastasis but rather in crosstalk with other signaling pathways and important molecular regulators. It is well known that HH signaling and *SOX* genes are in functional relationship during embryonic development[21,22]. However, little is known about their crosstalk in cancer cells. In this paper we addressed the question whether *SOX18* expression is under control of this signaling pathway in cervical carcinoma cell lines. Here we describetranscriptional regulation of the human *SOX18* gene in response to HH signaling and explored the possibilities for manipulation with its expression using specific agonists and antagonists of this signaling pathway. Also, we present data that will help in understandingof SOX18’s role in the regulation of tumorigenic features of cancer cells *in vitro*, in particular in regulation of cancer cell’s migration and invasion, as an important step in metastatic spreading.

## Materials and Methods

### Sequence analysis of the *SOX18* regulatory region

The MatInspector release professional 7.4 program was used to identify potential GLI transcription factor binding sites within *SOX18* regulatory region.

### Cell culture, transfection and co-transfections

HeLa (ATCC, CCL-2) cells were maintained in Dulbecco's Modified Eagle's medium (DMEM) supplemented with 10% fetal bovine serum (FBS) and 1% non-essential amino acids (NEAA) (all from Invitrogen, NY, USA), at 37°C in 5% CO_2._SiHa (ATCC, HTB-35) and Ca Ski (ATCC, CRL-1550) were maintained in Dulbecco's Modified Eagle's medium (DMEM) supplemented with 10% fetal bovine serum (FBS). Transfection experiments were carried out as previously described[23,24]. For co-transfection experiments, 10 μg of promoter reporter construct (892pCAT6) was co-transfected with 2 μg of either pcDNA4NLSMTGLI1, p4TO6MTGLI2 or pcDNA4/TO/GLI3 expression constructs[25,26]. β-gal and CAT assays were performed as previously described[27]. For imunocytochemistryanalysis, cells were cultured in 24 well dishes and GLI1, GLI2 or GLI3 were co-transfected with pEGFP-C1 (Clontech Laboratories, Mountain View, CA, USA) in ratio 9:1 using Lipofectamine (Invitrogene, NY, USA). For functional analysis of SOX18 protein, cells were transfected as previously described[23].

For modulation of HH signaling activity, cells were treated with 10 μM cyclopamine (Sigma-Aldrich, St.Louis, MO, USA), 10 μM tomatidine (Sigma-Aldrich, St.Louis, MO, USA), 10 μM purmorfamine (Sigma-Aldrich, St.Louis, MO, USA), or 20 μM GANT61 (Selleckchem, Houston, USA) for indicated periods of time.

### Western blot

Whole cell lysates (WCL) were prepared, proteins were separated and Western blot was performed as previously described[23]. Primary rabbit polyclonal antibodies against SOX18 (sc-20100; 1:1000) was purchased from Santa Cruz Biotechnology (Texas,USA), mouse monoclonal anti α-tubulin (CP06; 1:10000) was purchased from Calbiochem (Massachusetts, USA).

### RT-PCR and qRT-PCR analysis

Total RNA and cDNA syntesis were prepared as previously described[28]. RT-PCRs were performed using KAPA 2G Fast HotStart Ready Mix (Kapa Biosystems,Wilmington, MA, USA).

For quantitative PCR analysis, cDNAs were subjected to real time PCR using Power SYBR Green PCR Master Mix (Applied Biosystems®, Carlsbad, Germany) in 7500 Real Time PCR Systems (Applied Biosystems®, Carlsbad, Germany).All samples were measured in triplicate and the mean value was considered. The relative expression level of analyzedgenes was determined using comparative quantification algorithm where resulting ΔΔCt value was incorporated to determine the fold difference in expression (2^- ΔΔCt^). The sequence of primers used in this study was listed in Table 1.

**Table 1:** Primers used for RT-PCR and qRT-PCR.

Primer	Sequence
GAPDH F	5’-GGA CCT GAC CTG CCG TCT AG-3’
GAPDH R	5’-CCA CCA CCC TGT TGC TGT AG-3’
GLI1 F	5’-CAG TTA TGG GCC AGC CAG AGA-3’
GLI1 R	5’-TGG CAT CCG ACA GAG GTG AG-3’
GLI2 F	5’-AGC AGC AGC AAC TGT CTG AGT GA-3’
GLI2 R	5’-GAC CTT GCT GCG CTT GTG AA-3’
GLI3 F	5’-TCC AAC ACA GAG GCC TAT TCC AG-3’
GLI3 R	5’-CTC TTG TTG TGC ATC GGG TCA-3’
PTCH F	5’-ACC AGA ATG GGT CCA CGA CAA-3’
PTCH R	5’-AAA GTC TGA GGT GTC CCG CAA G-3’
SOX18 F	5’-TTC CAT GTC ACA GCC CCC TAG-3’
SOX18 R	5’-GAC ACG TGG GAA CTC CAG-3’

### Electrophoretic mobility shift assays (EMSA)

Nuclear extracts used in this study were prepared as described[29]. The oligonucleotides used in EMSA and supershift studies are listed in Table 2. Radiolabeling of probes and binding reactions were carried out as described[27]. In the supershift assays nuclear extracts were incubated with anti-SOX18 antibody for 20 minutes at room temperature before the probe was added. In competition assays, 100-fold molar excess of unlabeled competitor was included in the binding reaction.

**Table 2:** Oligonucleotides used in EMSA assays.

Primer	Sequence
SOX18FG1	5’-CAAGGGCCCTTGGGGGGCAGGGAGGACG-3’
SOX18RG1	5’-GGCGTCCTCCCTGCCCCCCAAGGGCCCTTG-3’
SOX18FG2	5’-GAGCCTCCCAGCGGGGGGCGGGGAACGGCAA -3’
SOX18RG2	5’-GGTTGCCGTTCCCCGCCCCCCGCTGGGAGGCTC -3’
SOX18FG3	5’-CCAGTTACTGCCCGGGGGTCCGACT -3’
SOX18RG3	5’-GGAGTCGGACCCCCGGGCAGTAACTGG -3’
SOX18FG4	5’-CGACTCCGTGGGTGGGTGGCAGCTCG -3’
SOX18RG4	5’-GGCGAGCTGCCACCCACCCACGGAGTCG -3’
SOX18FG5	5’CTTTCTTTCCCACCCGGGGGGTCTCT -3’
SOX18RG5	5’-GGAGAGACCCCCCGGGTGGGAAAGAAAG -3’
SOX18FG6	5’- GGGGGAGGTGGGGGGGCTGTGCGCGGGGGAGG -3’
SOX18RG6	5’- CCTCCCCCGCGCACAGCCCCCCCACCTCCC -3’
FGLI	5’- GGTTTAAGCTTCGTGGGTGGTCAC-3’
RGLI	5’-GTGACCACCCACGAAGCTTAAA -3’

### Proliferation assays

For analysis of HeLa cells proliferation rate upon HH signaling modulation, cells were seeded 3x10^5^ per 35 mm dish and grown for 1, 3 and 5 days in the presence cyclopamine or tomatidine and 1 and 3 days in the presence of purmorphamine. For analysis of SOX18 involvement in the regulation of HeLa cells proliferation, cells were transfected with either empty vector or pCISOX18*wt* or pCISOX18DN for 24 hours. Upon indicated duration of treatments or transfections viable cells were manually counted and proliferation curve was generated.

### Cell viability assay

Cells (2x10^3^) were seeded in 96-well plate, cultured overnight, treated with cyclopamine and tomatidine for 1, 3, 4 days and purmorphamine and DMSO for 1 and 3 days. Following the indicated treatments or 24 h after transient transfections with either empty vector or pCISOX18*wt* or pCISOX18DN, cell viability was assessed using 3-(4,5-dimethylthiazol-2yl)-2,5-diphenyltetrazolium bromide (MTT) assay. MTT solution was added to cell cultures at a final concentration of 0.5 mg/ml and cells were incubated for 1 h at 37°C. Subsequently, the medium was removed and the cells were lysed in DMSO. The conversion of MTT to formazan by metabolically viable cells was monitored by microplate reader at a wavelength of 620 nm. The experiment tests were done in double triplicates and repeated in at least three independent experiments.

### Wound-healing assay

3x10^5^ cells were plated in 35mm dish, grown to confluence and pre-treated with cyclopamine or purmorphamine and appropriate controls over night prior to the experiment. Confluent cell monolayer was scratched with 200μl tip, washed with serum-free medium to remove detached cells and finally fresh medium containing drugs was added. To monitor the effect of SOX18 overexpression on cell migration, cells were transfected withpCI, pCISOX18*wt* or pCISOX18DN 24 h prior to the wounding. Cell migration into the wounded area was monitored using DM IL LED Inverted Microscope (Leica Microsystems, Wetzlar, Germany) and closure of the gap distance was quantified using Leica Application Suite V4.3.0. The speed and the mode of cell migration were analyzed capturing two different parts of the wounded area from three independent experiments.

### Transwell migration and invasion assay

Corning Transwellpolycarbonate membrane cell culture inserts with 8.0 μm pores were uncoated for migration assay or coated with 50 ml BD Matrigel Basement Membrane Matrix (BD Biosciences) diluted 1: 3 with FBS-free DMEM for invasion assay. For both assays cells were transiently transfected 24h prior seeding with *wt* SOX18, DN SOX18 or corresponding empty vector as a control. Inserts were placed in 24 well plates with 10% FBS/DMEM in the lower chamber. In upper chamber, 10^5^ cells were directly seeded in 100 ml FBS-free DMEM. After 12 h culture, cells that had migrated to the bottom of the chamber membrane were fixed/stained with crystal violet solution (20% MtOH, 2% PFA, 0,5% crystal violet). For invasion assay, cells were allowed to migrate 24 h. After staining, cells on the upper surface were carefully removed using a cotton swab. Cells that migrated or invaded were visualized and photographed using a phase-contrast microscope DM IL LED Inverted Microscope (Leica Microsystems, Wetzlar, Germany) (x200 magnification). Five fields per filter were counted; the fields were randomly chosen from the top, bottom, left, right, and center positions of each filter. Three independent experiments were performed in duplicate wells.

### Immunocytochemistry

Cells were fixed in 4% paraformaldehyde for 20 min at room temperature (RT). Following permeabilization in 0.2% Triton X-100 and blocking in 1% bovine serum albumin (BSA), 10% normal goat serum and 0.1% Triton X-100 in PBS, cells were incubated in SOX18 antibody diluted (1:500) in 1% bovine serum albumin (BSA), and 0.1% Triton X-100 in PBS O/N at 4°C. The primary antibody was first labeled with biotinylated goat anti-rabbit IgG (Vector, Burlingame, CA, USA) for 1 h at RT in 1% BSA, followed by Cy3-streptavidin diluted 1:5000 (Jackson ImmunoResearch, West Grove, PA, USA) diluted in PBS for 1 h at RT. Nuclei were stained with 0.1 mg/ml diaminophenylindole-DAPI (Sigma-Aldrich, St. Louis, MO, USA). Samples were viewed by a Leica TCS SP8 confocal microscope and Leica Microsystems LAS AF-TCS SP8 software (Leica Microsystems, Wetzlar, Germany).

### Statistical analyses

Statistical analyses were performed with SPSS statistical software (version 20). The data represents means ± SEM from three to five independent experiments (indicated in figure legends). Statistical analyses were performed by student’s *t*-test andp value ≤ 0.05 was considered significant.

## Results

### GLI1 and GLI2, but not GLI3 are involved in the regulation of *SOX18* gene expression in cervical carcinoma cell lines

**Fig 1:**
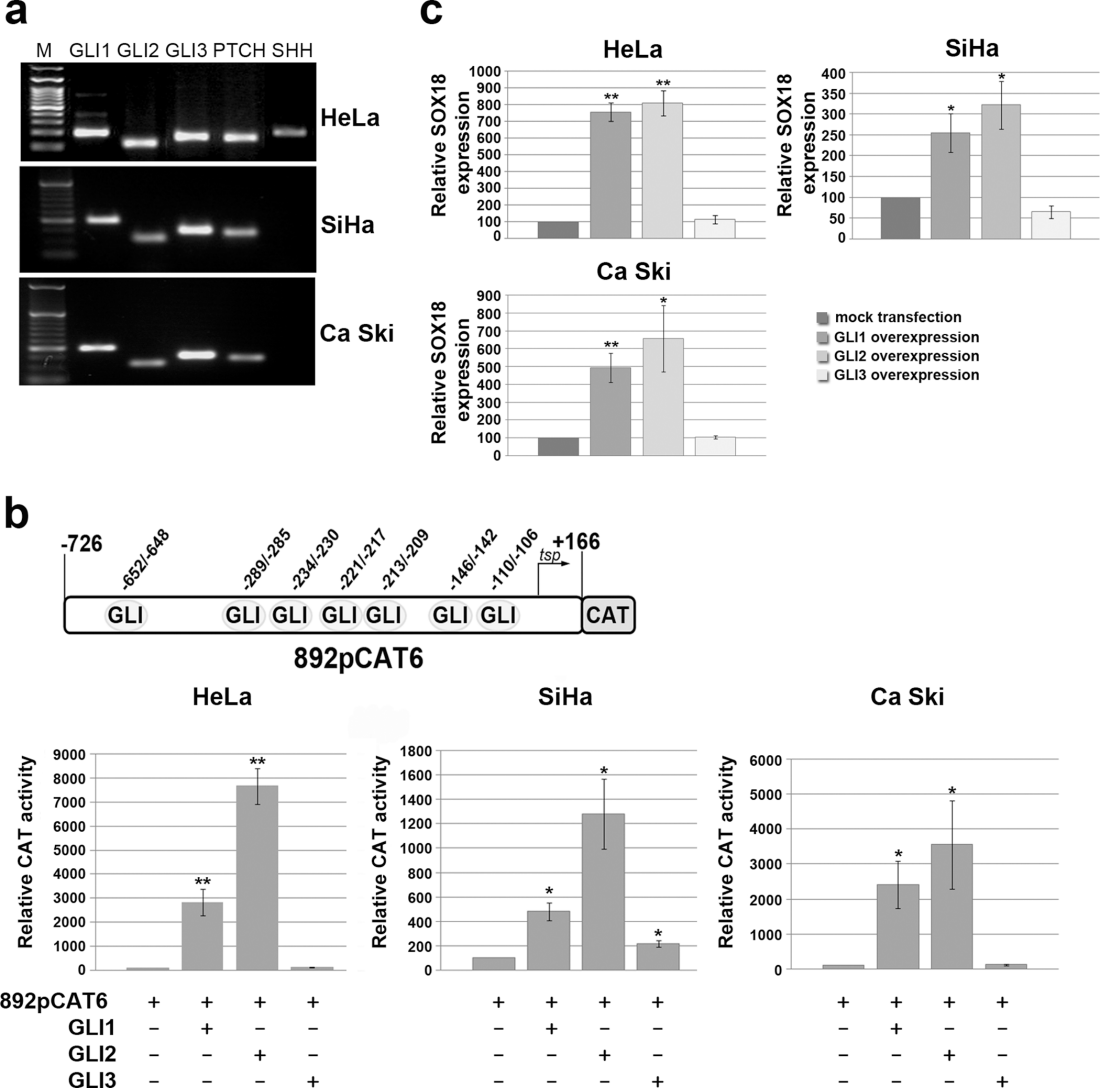
The role of GLI transcription factors in the regulation of *SOX18* promoter activity and *SOX18* endogenous expression. **a)** Expression analysis of selected HH signaling components in HeLa, SiHa and Ca Ski carcinoma cell lines.M-DNA ladder.**b)** Functional analysis of each GLI transcription factor overexpression on *SOX18* promoter activity. Schematic illustration of putative biding sites for GLI transcription factors within *SOX18* optimal promoter region represented by promoter construct 892pCAT6 is presented on upper panel. Normalized CAT activities were calculated as percentages of the corresponding reporter construct activity in cells co-transfected with empty pcDNA3.1 vector (which was set as 100%). Relative CAT activities were presented as the means ± SEM of at least four independent experiments. P values were calculated using Student’s *t*-test, *p ≤ 0.05, **p ≤ 0.01**c)**The effect of GLI’s overexpression on *SOX18* gene expression detected by qRT-PCR. Relative *SOX18* expression was presented as percentage of *SOX18* expression in mock transfected cells that was set as 100%. Results were presented as the means ± SEM of at least three independent experiments performed in triplicates. P values were calculated using Student’s *t*-test, *p ≤ 0.05, **p ≤ 0.01.

Previous studies showed that the expression of all the HH-signaling molecules were greatly enhanced in uterine cervical tumors, including carcinoma and its precursor lesions[30]. Therefore, we screened expression of components of HH signaling pathway in three cervical carcinoma cell lines: HeLa, SiHa and Ca Ski. We detected expression of HH effectors, GLI transcription factors, and PTCH receptor in all cell lines, while moderate SHH expression was detected only in HeLa cells (Fig 1A), suggesting constitutively active SHH signaling in this tumor-derived cell line. In order to test whether *SOX18* gene could be a direct target of HH signaling, we utilized MatInspector software and revealed seven putative binding sites for GLI transcription factors within previously defined optimal promoter of *SOX18* gene (Fig 1B, scheme in upper panel). Therefore, we performed functional analysis studying the effect of GLI transcription factors overexpression on the activity of *SOX18* promoter (represented with previously characterized SOX18construct 892pCAT6[24] in cervical carcinoma cells. After transient co-transfection of GLI1, GLI2 or GLI3 expression vector together with 892pCAT6 construct we showed that transcription factors GLI1 and GLI2 are potent activators of *SOX18*promoter in all cell lines(Fig 1B). Precisely, GLI1 overexpression led to approximately 30-, 4- and 25- fold induction,while GLI2 overexpression led to 80-, 12- and 35- fold induction of *SOX18* promoter activity in HeLa, SiHa and Ca Ski cells, respectively (Fig 1B). GLI3 overexpression had no effect in HeLa and Ca Ski, and moderately enhanced *SOX18* promoter activity in SiHa cells (Fig 1B). Taking together, both GLI1 and GLI2 significantly induced*SOX18* promoter activity in all three cervical carcinoma cell lines, with the strongest effect observed in HeLa cells.

Next, we tested whether this vast activation of promoter activity leads to up-regulation of endogenous *SOX18* gene expression. We showed that overexpression of GLI1 and GLI2 resulted in significant up-regulation of endogenous *SOX18* gene expression, while GLI3 remained ineffective in all analyzed cell lines (Fig 1C). Obtained results suggested that *SOX18* transcription was positively regulated by GLI1 and GLI2, at least in part, as a result of promoter activation. As a control experiment, we showed that GLI1 and GLI2 also up regulated PTCH transcription, as expected due to previous reports (S1B Fig)[31,32].

**Fig 2:**
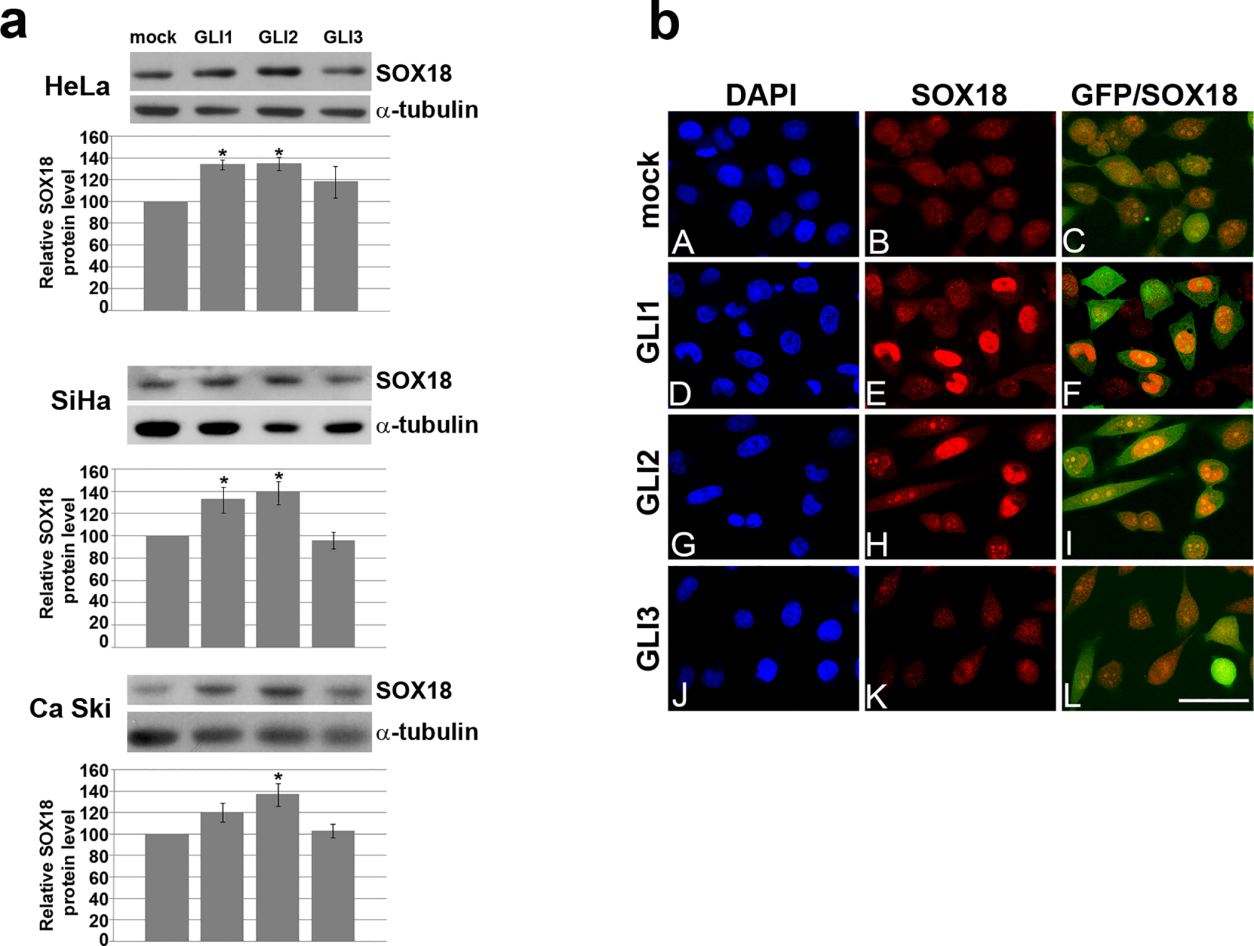
The effect of GLI’s overexpression on SOX18 protein level in cervical carcinoma cell lines. **a)** The effect of GLI’s overexpression on SOX18 protein leveldetected by Western blot. One representative blot was presented, and quantification of protein level was presented as histogram chart.α-tubuline was used as a loading control. The relative SOX18 protein level in HeLa, SiHa and Ca Ski cells upon transfection with GLI1-3 was calculated as a percentage of SOX18 level in mock transfected cells which was set as 100%. Data of three independent experiments are presented at histograms as the means ± SEM. Values of p≤0.05 are marked by *.**b)**The effect of GLI’s overexpression on SOX18 protein leveldetected by immunocytochemistry. Cells were cotransfected with EGFP-C1 (that was used as a marker of transfected cells) and eiherpcDNA-mock transfection (A-C), GLI1(D-F), GLI2 (G-I), or GLI3(J-L). Boxed regions in A-L are enlarged in the same figures. Cell nuclei were counterstained with DAPI (A, D, G and J). Scale bars: 50 μm.

Further, increase in SOX18protein level upon overexpression of GLI1 and GLI2 was confirmed by Western blot (Fig 2A) and immunocytochemistry (presented at Fig 2B for HeLa cells and within S2 Fig for SiHa and Ca Ski). These results confirmed that GLI1 and GLI2 are positive regulators of SOX18 in allanalyzed cervical carcinoma cell lines.Since the most prominent effect was detected in HeLa cells further experiments were continued using this cell line.

### GLI1 binds to three out of seven putative binding sites within *SOX18* promoter *in vitro*

**Fig 3:**
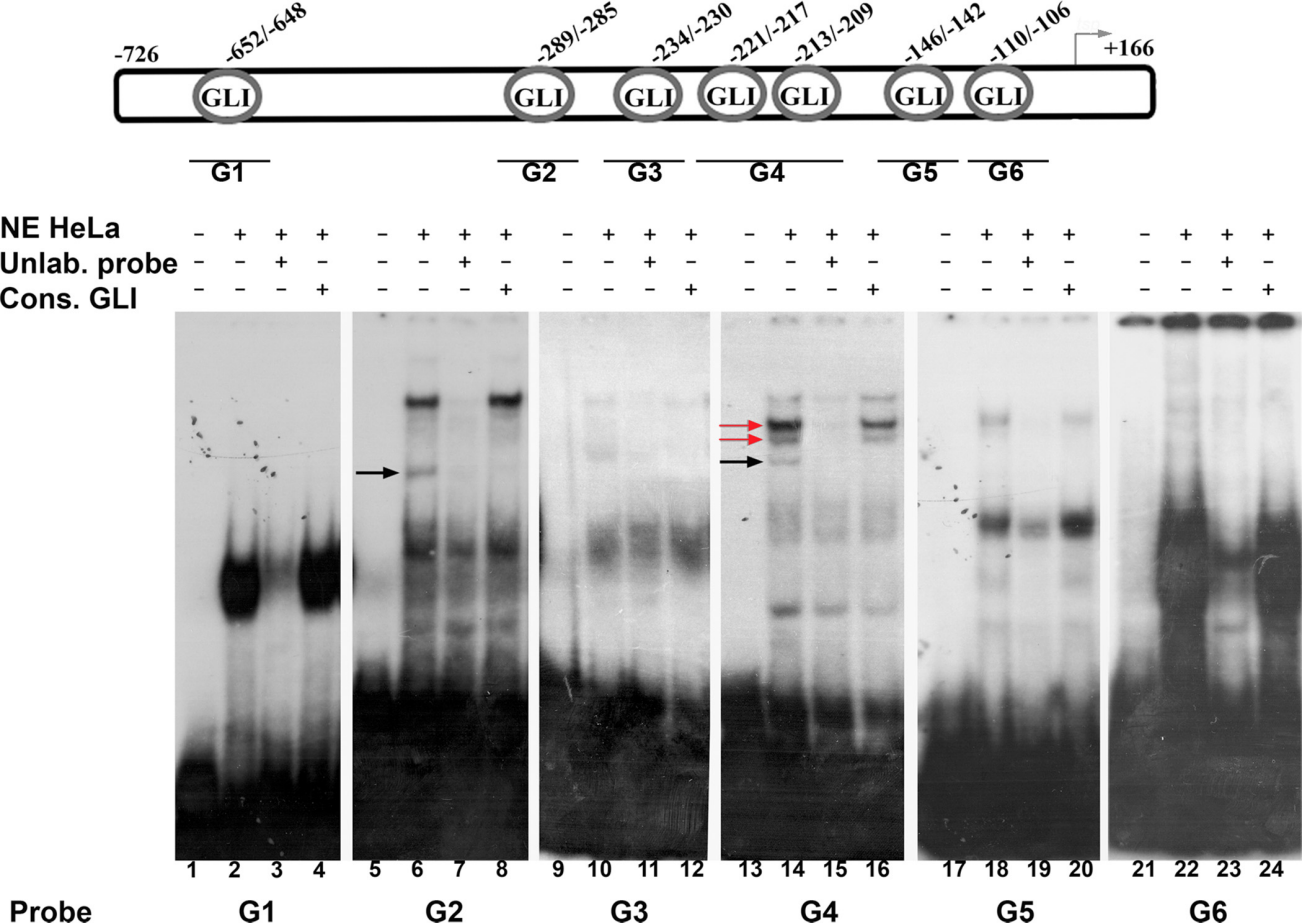
EMSA with six different DNA probes deriving from *SOX18* optimal promoter. Upper panel represents schematic illustration of putative GLI binding sites. Positions relative to *tsp* are indicated above scheme, and relative positions of corresponding probes are presented by lines and names below. Lower panel represents EMSA reactions; **NE HeLa** *-*HeLa nuclear extracts; **Unlab.probe**- unlabeled corresponding probe in 100-fold molar excess; **Cons.GLI**- unlabeled oligonucleotide probe with GLI consensus binding site in 100-fold molar excess. Completely faded complexes in competition reaction with GLI consensus probe are marked with black arrows, and partially faded with red arrows.

Using *in silico* analysis we identified seven putative GLI binding sites, within *SOX18* optimal promoter region. In order to determine which putative binding site is involved in binding of GLIs, we performed EMSA assay with six *SOX18* DNA probes (designated as probes G1 to G6) and nuclear proteins obtained from HeLa cells. DNA probes G1, G2, G3, G5 and G6 contain single GLI binding site, while probe G4 has two, closely positioned putative GLI binding sites (Fig 3, upper panel, schematic illustration). Nuclear proteins isolated from HeLa cells bound to all six DNA probes and form specific protein-DNA complexes (Fig 3, lanes 2, 6, 10, 14 and 22 compared to free probes in lanes 1, 5, 9, 13 and 21). Specificity of formed complexes with each probe was tested in competition reaction with 100-fold molar excess of corresponding unlabeled probe (Fig 3, lanes 3, 7, 11, 15 and 23). In order to elucidate whether GLI proteins participate in protein-DNA complex formation, we performed competition with oligonucleotide probe containing GLI consensus binding site. If GLI proteins are present in formed complexes, competition with GLI consensus probe will lead to fading of complexes due to binding of GLI proteins predominantly to their consensus binding sites. In competition reaction with 100-fold molar excess of unlabeled GLI consensus probe, we observed considerable fading of protein-DNA complexes formed by probes G2 and G4 (Fig 3, lanes 8 and 16; faded complexes are marked by arrows), and absence of competition with other probes (Fig 3, lanes 4, 12, 20 and 24). This result indicates that GLI proteins are able to bind within probes G2 and G4.

**Fig 4:**
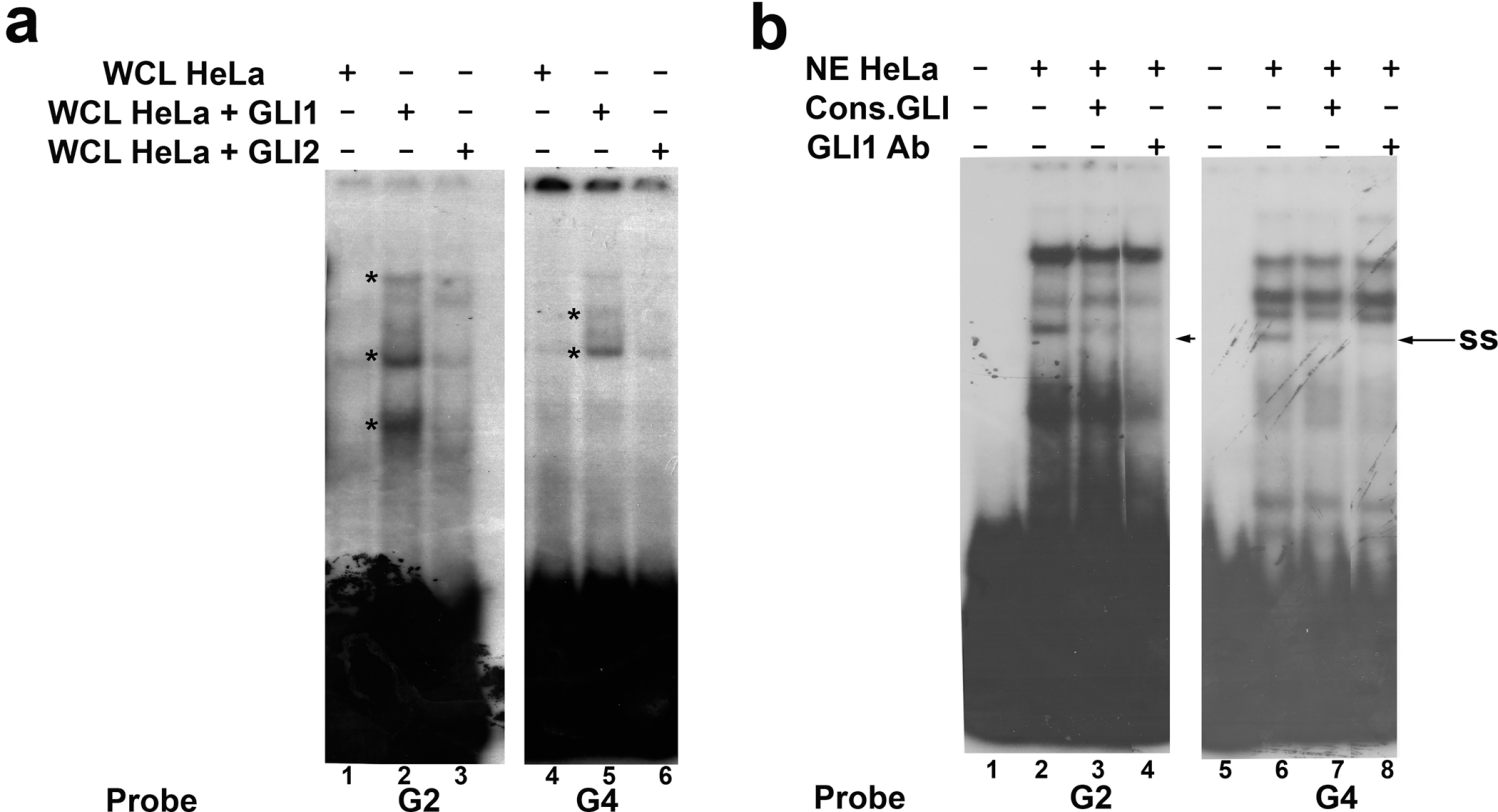
EMSA with “enriched” proteins and supershift assay. **a)** EMSA with whole cell lysates prepared from HeLa cells transfected with pcDNA3.1 empty vector (WCL HeLa) or cells transfected with either GLI1 (WCL HeLa+GLI1) or GLI2 expression vector construct (WCL HeLa+GLI2). **b)**Supershift assay with anti-GLI1 antibody (GLI1Ab). “Enriched” complexes are marked by asterisks,supershifts i.e. fading of complexes are marked by arrows and ss.

Since we showed that GLI1 and GLI2 are involved in up-regulation of *SOX18* expression, we performed EMSA assay with proteins isolated from HeLa cells transfected with GLI1 or GLI2 expression constructs, using probes G2 and G4. Compared to the binding of proteins isolated from mock transfected cells, we detected increased binding of proteins “enriched” with GLI1 (Fig 4A, lanes 2 and 5, comparing with lanes 1 and 4, increased binding is marked by asterisk), while overexpression of GLI2 did not significantly changed the intensity of formed complexes (Fig 4A, lanes 3 and 6, comparing with lanes 1 and 4). Therefore, we concluded that GLI1 contributes to protein-DNA complexes formation with probes G2 and G4. To further validate involvement of GLI1 in these complexes we performed EMSA supershift assay with specific antibodies against GLI1 protein. After addition of anti-GLI1 antibodies (Fig 4B, lanes 4 and 8), we observed fading of the same complex as in competition reaction with consensus GLI oligonucleotide probe (Fig 4B, lanes 3 and 7). Fading of protein-DNA complexes in supershift reaction has already been shown in several reports and was considered as a confirmation of specific protein presence[27]. Taken together, we showed that GLI1 transcription factor has the ability to bind*in vitro*to the sequences located -289 to -285 and -221 to -209 relative to *tsp* within *SOX18* optimal promoter region.

### Inhibition of HH signaling impairs cells proliferation, viability and migration and reduces *SOX18* expression

**Fig 5:**
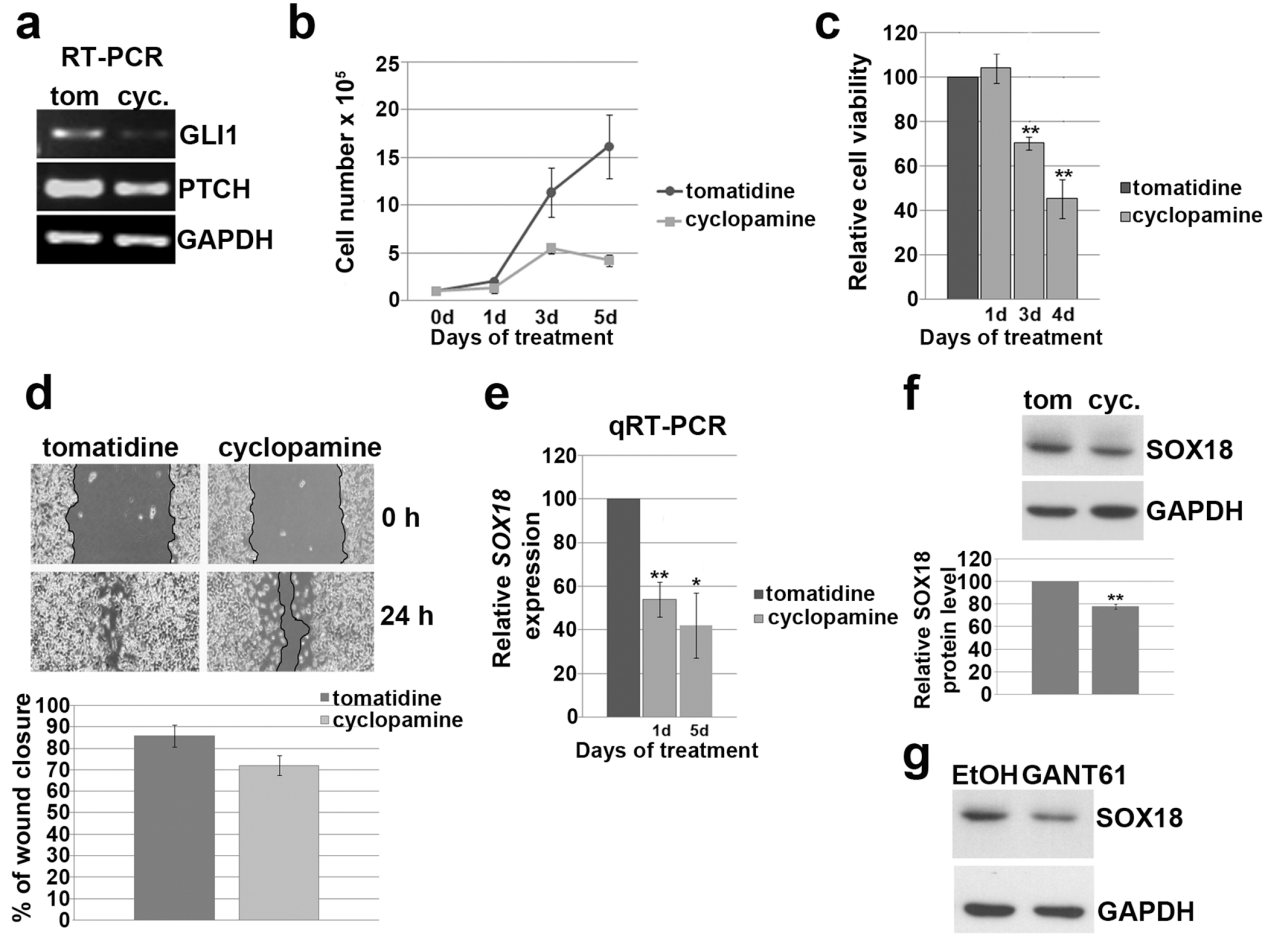
The effect of HH pathway inhibition on proliferation, viability, migration and *SOX18* expression in HeLa cells. **a)** The inhibitory effect of cyclopamine on GLI1 and PTCH expression. **b)** Proliferation curve of HeLa cells. Cells were treated with 10μM cyclopamine or tomatidine as a negative control, and counted after 1, 3 and 5 days of continuous treatment. Results were presented as the means ± SEM of at least three independent experiments. **c)** MTT viability assay performed after 1, 3 and 4 days of treatment with 10 μM cyclopamine or tomatidine. Relative cell viability was calculated as a percentage of HeLa cells viability after tomatidine treatment that was set as 100%. Results were presented as the means ± SEM of at least three independent experiments. P values were calculated using Student’s *t*-test, *p ≤ 0.05, **p ≤ 0.01.**d)** The effect of cyclopamine on cell’s migration, wound-scratch migration assay. Cells migration was quantified 24 h after scratching in constant presence of cyclopamine or tomatidine,by measuring the difference in gap closure where gap wide at 0 h was set as 100%. Results were presented as the means ± SEM of at least three independent experiments. **e)** Relative *SOX18* expression after cyclopamine treatment detected by qRT-PCR. Relative *SOX18* expression was presented as percentage of *SOX18* expression in cells treated with tomatidine that was set as 100%. Results were presented as the means ± SEM of at least three independent experiments performed in triplicates. P values were calculated using Student’s *t*-test, *p ≤ 0.05, **p ≤ 0.01.**f)** The effect of cyclopamine on SOX18 protein level. Proteins were isolated after three independent treatments together with adequate controls, followed by Western blot. One representative blot was presented. α-tubuline was used as a loading control. The relative SOX18 protein level in HeLa cells upon treatment with cyclopamine was calculated as a percentage of SOX18 level in cells trated with tomatidine which was set as 100%. Data of three independent experiments are presented at histograms as the means ± SEM. Values of p≤0.01 are marked by **.**g)** The effect of GANT61 on SOX18 protein level. Proteins were isolated after three independent treatments together with adequate controls, followed by Western blot. One representative blot was presented. α-tubuline was used as a loading control.

Canonical HH signaling involves HH ligands and activation of GLI transcription factors implicated in regulation of their target genes. However, plenty of evidencehas been presented showing that GLI transcription factors could be activated in HH-ligand independent manner by various cytokines and chemokines[33]**.** In order to test whether activation of *SOX18* expression by GLI1 and GLI2 is linked to canonical HH pathway activity, we treated HeLa cells with cyclopamine. Cyclopamine is naturally occurring, small-molecule specific inhibitor of HH signaling pathway[34,35]. This inhibitory effect is mediated by direct binding to Smoothened (SMO) receptor[36] that in consequence leads to impaired signaling transduction. Tomatidine, a structural analogue of cyclopamine that does not inhibit HH signaling, was used as control. The inhibitory effect of cyclopamine was tested monitoring the changes in transcription levels of GLI1 and PTCH, two direct markers of HH pathway activity. As presented, both genes were down regulated and that was considered as the evidence of efficient inhibition of HH pathway (Fig 5A). We tested the effect of cyclopamine treatment on cell’s proliferation and viability and found that the addition of cyclopamine significantly reduced cell proliferation up to approximately 75% after 5 days of treatment (p = 0.025) and viability up to 45% after 4 days of treatment respectively (p = 0.003) (Fig 5B and 5C).

Since the ability of cancer cells to migrate is closely associated with their capacity to colonize distant organs, we tested migratory potential of HeLa cells, in response to HH pathway inhibitor, using wound-scratch assays. We detected that HeLa cells treated with cyclopamine were slower in closing the scratched area than control cells (Fig 5D), indicating that inhibition of HH signaling in HeLa cells also impairs process of cells migration.

Giving that cyclopamine exhibited inhibitory effect on HeLa cell’s viability and migration, we finally tested whether this inhibition of HH signaling leads to alteration in *SOX18* expression. As presented at Fig 5E cyclopamine treatment caused decrease in *SOX18* gene expression by approximately 50%, reveled by qRT-PCR. The inhibitory effect of cyclopamine was also observed on protein level (Fig 5F). Since we showed that GLI1 and GLI2 up-regulated*SOX18*expession, we employed in our analysis GANT61, inhibitor of both GLI1 and GLI2. By treatment with GANT61 we targeted HH signaling downstream of SMO receptor. Treatment with GANT61 led to reduction in SOX18 protein level (Fig 5G). We clearly demonstrated that inhibition of HH signaling, using both cyclopamine and GANT61, inhibited *SOX18* expression, and that this inhibition was largely mediated through GLI1 and GLI2.

### Activation of HH signaling promotes cells proliferation, viability and migration and up-regulates *SOX18* expression

In order to enhance HH signaling activity in HeLa cells we have used purmorphamine, a small-molecule agonistthat activates the HH pathway by targeting SMO[37].

**Fig 6:**
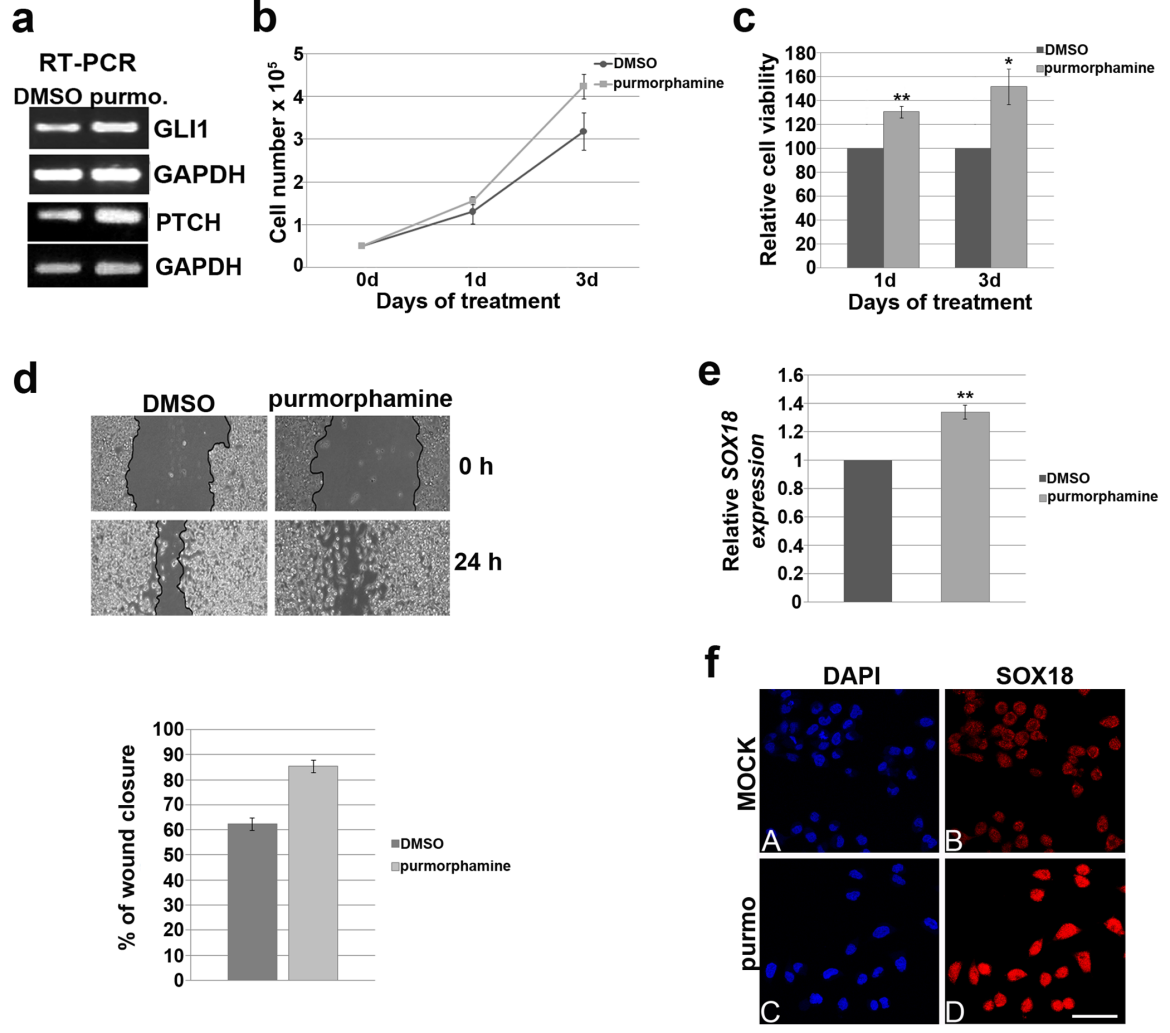
The effect of HH pathway activation on proliferation, viability, migration and *SOX18* expression in HeLa cells. **a)** Stimulatory effect of purmorphamine on GLI1 and PTCH expression. **b)** Proliferation curve of HeLa cells. Cells were treated with 10μM purmorphamine or DMSO as a control, and counted after 1 and 3 days. Results were presented as the means ± SEM of at least three independent experiments. **c)** MTT viability assay performed after 1 and 3 days of treatment with 10μM purmorphamine or DMSO. Relative cell viability was calculated as a percentage of HeLa cells viability after DMSO treatment that was set as 100%. Results were presented as the means ± SEM of at least five independent experiments. P values were calculated using Student’s *t*-test, *p ≤ 0.05, **p ≤ 0.01.**d)** The effect of purmorphamine treatment on cell migration, wound-scratch migration assay. Cells migration was quantified 24 h after scratching in constant presence of purmorphamine or DMSO, by measuring the difference in gap closure where gap wide at 0 h was set as 100%. Results were presented as the means ± SEM of at least three independent experiments. **e)** Relative *SOX18* expression after purmorphamine treatment detected by qRT-PCR. Relative *SOX18* expression was presented as percentage of *SOX18* expression in cells treated with DMSO that was set as 100%. Results were presented as the means ± SEM of at least three independent experiments performed in triplicates. P values were calculated using Student’s *t*-test, *p ≤ 0.05, **p ≤ 0.01.**f)** The effect of purmorphamine treatment on SOX18 protein leveldetected by immunocytochemistry. Cell nuclei were counterstained with DAPI (A, and C). Scale bars: 50 μm.

First, we confirmed stimulatory effect of purmorphamine in HeLa cells by analysis of GLI1 and PTCH expression and detected up-regulation of their expression upon treatment (Fig 6A). After 3 days of treatment, purmorphamine increased cell proliferation and viability by approximately 30% and 50%, respectively (Fig 6B and 6C). Further, as presented at Fig 6D, we noticed differences between migratory potential of cells treated with purmorphamine versus control cells. We observed that treated cells migrated faster than control cells. Also, the motility of treated cells could be described as a confluent front, while purmorphamine treated cells were able to move individually into the empty scratched area. This result suggests that activated HH signaling possibly promotes single cell motility in HeLa cells.

Regarding *SOX18* expression, purmorphamine treatment led to up-regulation of *SOX18* expression by approximately 35% (Fig 6E). Elevated SOX18 protein level upon purmorphamine treatment was also detected by immunocytochemistry (Fig 6F).

### Overexpression of *SOX18* has no influence on proliferation and viability, but promotes migration and invasion of HeLa cells

**Fig 7:**
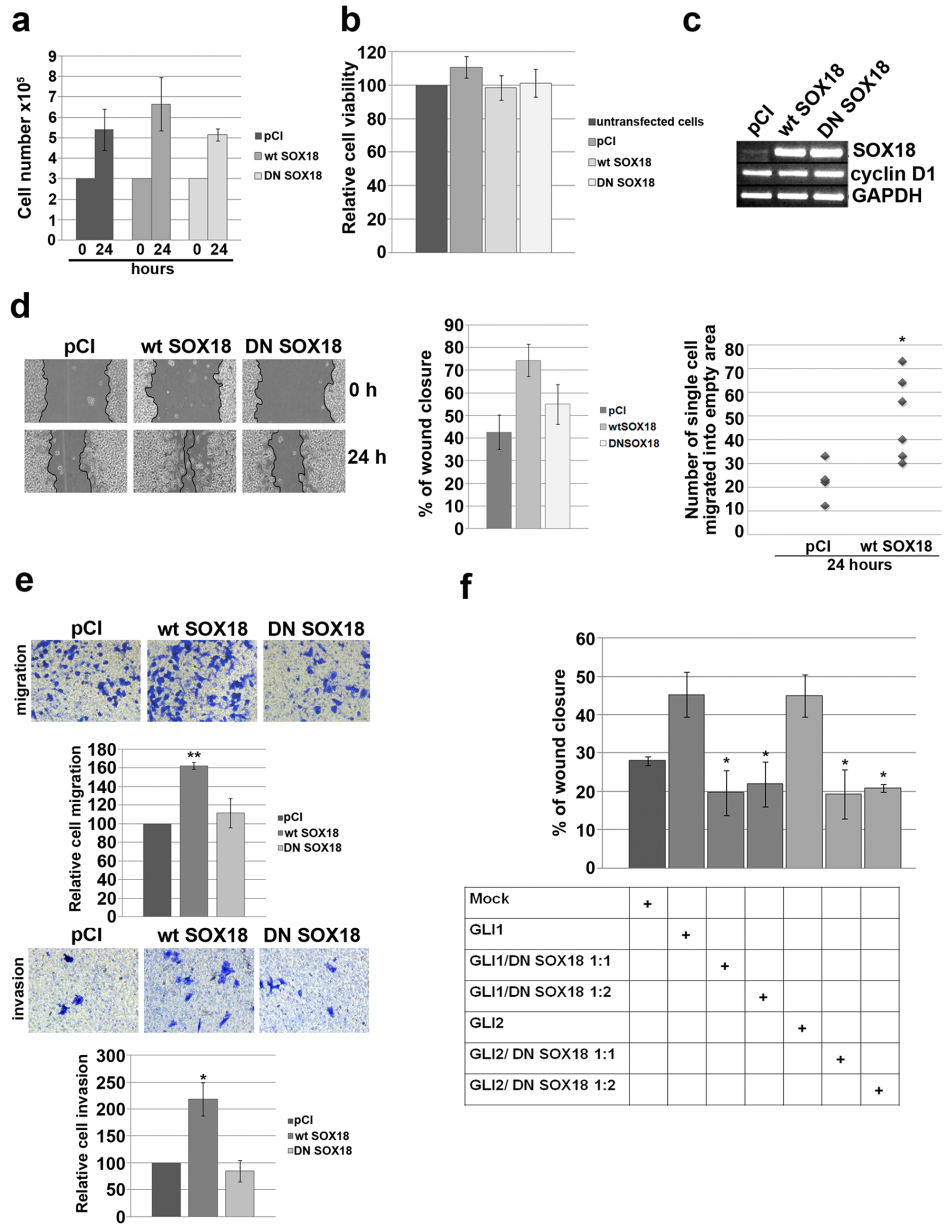
The role of SOX18 in the regulation of HeLa cells proliferation, viability and migration. **a)** Proliferation chart. HeLa cells were seeded day prior to transient transfection with empty pCI vector, *wt* or DN SOX18 expression constructs and counted 24 h after transfection. Results were presented as the means ± SEM of at least four independent experiments. **b)** MTT viability assay. HeLa cells were transiently transfected while seeded in microplate and MTT test was performed 24 h later. Relative cell viability was calculated as a percentage of HeLa cells viability without transfection that was set as 100% Results were presented as the means ± SEM of at least four independent experiments. **c)**Semiquantitative RT-PCR. Cells were transiently transfected with empty pCI vector, *wt* or DN SOX18 expression constructs followed by RNA isolation and RT-PCR analysis for the expression level of *cyclin D1*. The level of *wt* or DN SOX18 transcription upon transfection was also evaluated. **d)** The effect of *wt* or DN SOX18 overexpression on HeLa cell’s migration, wound-scratch migration assay. Representative images of cells migration were presented at left panel. Graphs presented at right panels quantify the migration of transfected cells 24 h after scratching. The changes in migration distance were quantified by measuring the difference in gap closure where gap wide at 0 h was set as 100%. Results were presented as the means ± SEM of at least three independent experiments. The differences in number of single cells that migrated into the empty area was measured by counting the number of single cells in empty scratched area in two different fields in at least three independent experiments and presented as scatter chart. P values were calculated using Student’s *t*-test, *p ≤ 0.05, **p ≤ 0.01.**e)** Transwell migration and invasion assays on HeLa cells transfected with empty pCI vector, *wt* SOX18 or DN SOX18. Representative images of transwell migration/invasion assays were presented.The relative change in cells migration/invasion was calculated as a percentage of HeLa cells migration/invasion in mock transfection that was set as 100%. Cells were counted from five fields and averages were calculated. Results were presented as the means ± SEM of at least three independent experiments performed in duplicates and P values were calculated using Student’s *t*-test, *p ≤ 0.05, **p ≤ 0.01.**f)** The influence of DN SOX18 overexpression on GLI1/GLI2-mediated HeLa cell’s migration, wound-scratch migration assay. Graph quantifies the migration of transfected cells 24 h after scratching. Table describes combination of expression vectors used in each experiment. The changes in migration distance were quantified by measuring the difference in gap closure where gap wide at 0 h was set as 100%. Results were presented as the means ± SEM of at least three independent experiments. P values were calculated using Student’s *t*-test comparing group 1 (cells transfected with GLI1 or GLI2) with group 2 (cells co-transfected with GLI1 or GLI2 together with DN SOX18);*p ≤ 0.05, **p ≤ 0.01.

In previous experiments we showed that *SOX18* expression in HeLa cells in under positive control of HH signaling pathway. To understand the implication of *SOX18* up-regulation upon HH signaling activation we addressed the question whether SOX18, as a regulatory protein, is involved in the control of proliferation, viability, migration and invasion of HeLa cells. Since SOX proteins, in general, act in functionally redundant manner, we decided to use dominant-negative strategy in order to test function of SOX18 protein. For that purpose we overexpressed either *wild type* (*wt* SOX18) or dominant-negative (DN SOX18) form of SOX18 proteins (expression constructs previously described)[23] and no significant changes were detected in proliferation and viability of HeLa cells (Fig 7A and 7B). This was opposite to our previous result showing that modulation of HH activity influence HeLa cells proliferation, which led us to assume that HH regulation of HeLa cell’s proliferation and viability is not mediated by SOX18. Since it has been reported that cell cycle regulator cyclin D1 expression is considerably increased during HH pathway activation[38–40], we analyzed cyclin D1 expression upon overexpression of either *wt* or DN form of SOX18 protein (Fig 7C). We could not detect any evident change in expression of this cell cycle regulator in response to *wt* SOX18 or DN SOX18. This result, again, excluded the involvement of SOX18 in the regulation of HeLa cell’s proliferation.

On the other hand, wound-scratch assay revealed that upon overexpression of *wt*SOX18, cells migrated faster than both control cells and cells transfected with DN SOX18 (Fig 7D). Also, like it was shown for purmorphamine treatment, cells with overexpressed *wt* SOX18 protein have a tendency to move individually into the empty scratched area compared to the control (Fig 7D, scatter chart). The average number of single cells migrated into the scratch area, 24 h after scratching, was significantly higher in cells transfected with *wt* SOX18 (on average 49 cells per gap) than in mock transfection (on average 24 cells per gap) (p = 0.017). These results imply that *SOX18* overexpression influences the mode of migration causing a switch from cohesive to single cell motility. Cell migration and invasion capabilities were also measured *in vitro* using uncoated or Matrigel-coated transwell inserts. As presented at Fig 7E, *wt*SOX18 overexpression significantly induced migration (1.6-fold, p = 0.004) and invasion rate (2.2-fold, p = 0.048) of HeLa cells. Average number of cells that migrated or invaded the matrigel in *wt*SOX18 overexpresing samples were 365 and 31 respectively, so in general invasion rate was approximately 10-fold lower. Together, these results indicated that SOX18 plays an important role in regulating the migration and invasion activities of HeLa cells.Finally, we have tested whether DN SOX18 overexpression could, to some extent, overcome GLI1/GLI2-mediated HeLa cell’s migration. As presented at Fig 7F, we have transfected HeLa cells with either GLI1 or GLI2 expression constructs alone, or together with increasing amount of DN SOX18 expression construct. Overexpression of both GLI1 and GLI2 have promoted HeLa cell’s migration, while addition of DN SOX18 led to significant reduction of GLI1 and GLI2-induced migration for approximately 20% (p = 0.022 and p = 0.028, respectively). However, we have not detected a dose-dependent inhibition. Taking together, migration of HeLa cells during activated HH signaling pathway is, at least in part, regulated by SOX18 transcription factor.

### Overexpression of *SOX18* in HeLa cells leads to down-regulation of GLIs transcription

**Fig 8:**
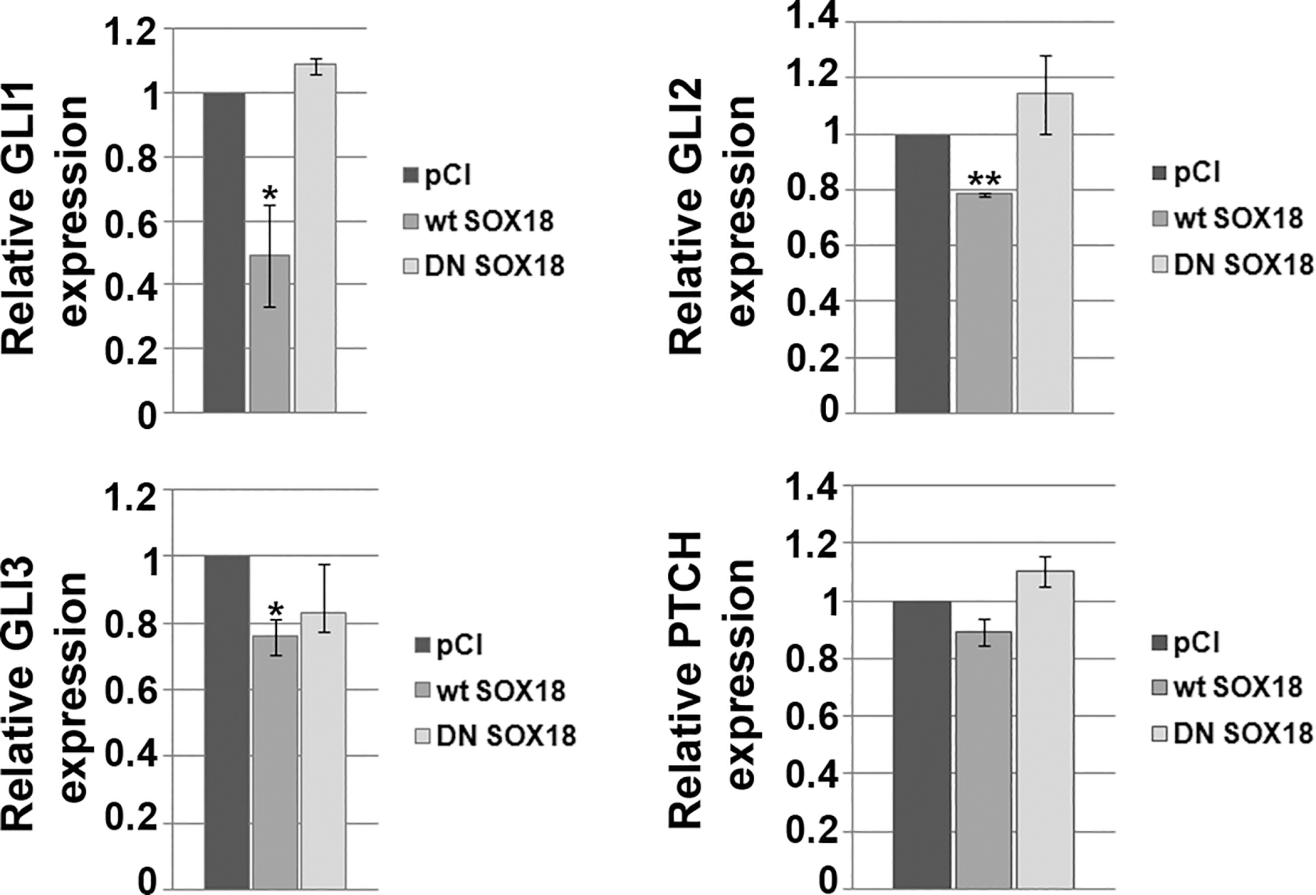
The role of SOX18 in the regulation of GLI1-3 and PTCHexpression. HeLa cells were transiently transfected with either empty pCI vector or *wt*SOX18 or DNSOX18 and the effect of their overexpression on GLI1-3 and PTCH genes expression was analyzed by qRT-PCR. Relative gene expression was presented as percentage of gene expression in cells transfected with empty, pCI vector that was set as 100%. Results were presented as the means ± SEM of at least three independent experiments performed in triplicates.P values were calculated using Student’s *t*-test, *p ≤ 0.05, **p ≤ 0.01.

As presented in this paper, *SOX18* gene expression is dependent on HH signaling pathway activity in HeLa cells. We were interested in studying potential regulatory crosstalk between SOX18 and GLI transcription factors and PTCH receptor. Therefore, we transiently overexpressed either *wt* or DN SOX18 protein and analyzed their effects on expression level of GLI1-3 and PTCH genes. As presented at Fig 8, *wt* SOX18 overexpression resulted in down-regulation of GLI1, GLI2 and GLI3, with most prominent effect on GLI1 expression that was decreased approximately 50%. Regarding PTCH expression, no significant change occurred upon *wt* SOX18 overexpression. In parallel, we analyzed the effect of DN SOX18 overexpression and, as expected, dominant-negative form of SOX18 protein remained ineffective. Taking together, these results suggest a negative feed-back mechanism involved in crosstalk between HH signaling pathway and SOX18 in HeLa cells.

## Discussion

SOX family of transcription factors may act as oncogenes, tumor suppressors, or both depending on the cellular context, and can be activated or inactivated through a variety of genetic and epigenetic mechanisms[41]. They contribute to the malignant phenotype through regulation of numerous processes in cancer cells including cell proliferation, apoptosis, survival, invasion, migration, differentiation, stemness, senescence, and angiogenesis[42–45].

Although there is much evidence showing functional relationship between Hedgehog pathway, in particular Sonic hedgehog and SOX transcription factors during embryonic development, scarce data are available regarding their crosstalk in cancer cells. It has been reported that SHH signaling can maintain *SOX9* overexpression in skin tumors[46] and colorectal cancer[47]. *SOX2*is regulated by HH signaling where transcriptionfactorsGLI1 and GLI2 directly bind to the proximal promoter region of SOX2 in primary melanoma cells[48].Also, human *SOX14* expression is GLI1 dependent in U87MG cells and SHH dependent in U87MG and HepG2 cells[49].

Until now, crosstalk between *SOX18* and HH signaling pathway has been reported in nonmalignant background[50,51]. Our findings link regulation of *SOX18* transcription with HH signaling and its final effectors, GLI transcription factors in cervical carcinoma cell lines.

Changes in the activity of HH signaling pathway are being recognized as an important oncogenic switch in many epithelial tumors. Several studies have reported correlation between HH pathway activity and its role in cervical carcinogenesis. It has been shown that HH signaling pathway is activated in both cervical squamous cell carcinoma and adenocarcinoma and also in cervical intraepithelial neoplasia [30,52]. HH signaling proteins, PTCH, SMO, and GLI2 seem to have prognostic value in cases with residual carcinoma, local recurrences, and for GLI2 distant relapses [52]. Also, there are data presenting a role of HH pathway in repopulation after chemoradiation of cervical carcinoma patients [53]. In addition, it has already been shown that HH pathway influence cervical cancer cell proliferation, survival and migration *in vitro*[54].

In this paper, we have shown that GLI1 and GLI2 act as important positive regulators of *SOX18* expression in HeLa, SiHa and Ca Ski cells. It is important to point out that GLI1 and GLI2 could be induced by other factors, like TGF-β, independently from SMO receptor and HH pathway activity[55,56]. Therefore, in order to elucidate the involvement of canonical HH pathway in the regulation of *SOX18* gene expression, we have used a specific SMO inhibitor, cyclopamine and showed that this small molecule inhibitor is able to reduce *SOX18* gene expression. Moreover, *SOX18* expression was successfully inhibited by GANT61,an inhibitor forGLI1as well as GLI2-induced transcription. The reduction in *SOX18* expression induced by HH inhibitors revealed that its expression, at least in part, depends on active canonical HH signaling in HeLa cells.

Over the years it became increasingly clear that SOX18 protein plays an important role in promoting tumor angiogenesis and therefore emerged as a promising potential target in antiangiogenic tumor therapy. Recently, two studies have been published revealing the high expression of SOX18 not only in blood and lymphatic vessels, but also in nucleus of cancer cells of invasive breast and ovary carcinomas[20,57]. Now, it becomes evident that the expression of *SOX18* gene in tumors is not restricted simply to the endothelium of accompanying blood and lymphatic vessels, and that its role in tumor development and progression might go beyond regulation of tumor angiogenesis and lymphangiogenesis. Although the concept of targeting SOX18 as a part of antitumor/antiangiogenic therapy is well known for several years, it is evident that the achievement in this field has been incomplete. Here, we presented first data showing that *SOX18* expression could be targeted by HH pathway inhibitors. It is important to point out that HH signaling is mainly inactive in normal adult cells, and becomes reactivated in several cancers, so using HH inhibitors could assure selective approach in modulating SOX18 level.

In order to get further insight into the specific role of *SOX18* up-regulation in response to HH pathway activation, we analyzed whether SOX18 transcription factor is involved in regulation of cell’s proliferation, viability, migration and invasion. We could not detect any changes in HeLa cell’s proliferation and viability upon ectopic overexpression of *wt* SOX18 or its dominant-negative counterpart, even though modulation of HH pathway in HeLa cells affected these processes. Since the mechanism by which HH signaling cascade regulates proliferation is now relatively well understood and involves the activation of cyclins and cyclin dependent kinases[58], we analyzed the effect of SOX18 on cyclin D1 expression and again excluded the role of SOX18 in the regulation of HeLa cell’s proliferation. Although Young et al. reported that knock-out of *SOX18* expression in MCF-7 cells results in an abrogation of cancer cell proliferation[19], here we confirmed results previously reported by Pula et al., that *SOX18* expression does not correlate with cancer cell proliferation[20].

On the other hand, we detected that SOX18 transcription factor could play important role in migration of cancer cells *in vitro*. We detected promoting effect of SOX18 on migration that is opposite to its effect on proliferation. Although highly proliferative tumors are often highly invasive, there are examples showing that these processes could exclude each other, mostly within various tumors of the central nervous system[59–61]. Understanding of the relationship between proliferation and migration is necessary for development of therapies aimed to inhibit both processes. Also, our results imply that SOX18 does not only increase cell motility, but also alters the mode of cell migration. In *wt*SOX18-overexpressing HeLa cells we observed tendency to switch from cohesive to single cell motility. Literature data demonstrated that, during dissemination, tumor cells could migrate as individual cells or in a group [62]. In many tumors, both types of dissemination can be present at the same time [63]. Changes in the mode of cell motility affect metastasis. It has been shown that the mode of migration governs the haematogenous or lymphatic spread: single cell motility increased the ability of cells to enter into the bloodstream while cohesive motility reduced cell entrance into the bloodstream but allows the lymphatic spread[64]. Considering these results we postulate that SOX18 overexpression could be involved in promotion of blood-borne metastasis. Finally, we demonstrated that inhibition of SOX18 function, using dominant-negative approach, inhibits to some extent GLI1/GLI2-mediated migration of cervical carcinoma cells *in vitro*. GLI transcription factors activity has been shown to promote the growth, migration and invasion of several cancer types [65–68]. The exact mechanisms by which GLI transcription factors achieve their pro-migratory effect are largely described in a range of cancer types and, among others, include involvement of variousmatrix metalloproteinases[65,67]. Inhibition of SOX18 led to reduction of GLI1/GLI2-mediated migration to some extent, but prevention of migration was not expected due to activity of other important mechanisms that are responsible for the regulation of cell migration. However, it is important to point out that one of matrix metalloproteinases (MMP7) has been previously identified as a SOX18 target gene in endothelial cells [69]. Hypothetically, SOX18 pro-migratory properties could be achieved through modulation of expression of this group of proteins.

So far, proposed targeting of SOX18 function included strategy of using dominant- negative SOX18 protein[19,70], but potential application of this approach is not fully elaborated. On the other hand, progress in the discovery of novel HH signaling inhibitors has provided many opportunities for developing novel cancer therapeutic strategies. There are three major targeting sites for HH signaling inhibitors identified so far: HH molecules, SMO receptor and GLI transcription factors. We have shown that SMO inhibitor cyclopamine and GLI inhibitor GANT61 are both able to down regulate *SOX18* expression in HeLa cells, opening a new field of potential manipulation with this gene expression in cancer. As in development of any targeted therapy, there are some challenges that prevent wider use of HH signaling inhibitors in clinics. These challenges include lack of basic understanding of molecular mechanisms by which HH signaling mediates carcinogenesis. Therefore, we believe that identification of *SOX18* as a novel target of Hedgehog signalingwill contribute to better understanding of these processes opening possibilities for novel targeted approaches.

## Supporting Information

S1 Fig Transient transfection of HeLa, SiHa and Ca Ski cells with GLI transcription factors. **a)** Overexpression of each GLI transcription factor in HeLa, SiHa and Ca Ski cells. **b)** The effect of GLIs overexpression on *PTCH* gene in HeLa cells.
(TIF)

S2 Fig The effect of GLI1 and GLI2 overexpression on SOX18 protein level in SiHa and Ca Ski cells, detected by immunocytochemistry. (TIF)
