# Supplementary figures and images for: SOX18 Is a Novel Target Gene of Hedgehog Signaling in Cervical Carcinoma Cell Lines
Source: PLoS One. 2015 Nov 20;10(11):e0143591. doi: 10.1371/journal.pone.0143591 (PMC4654472; doi:10.1371/journal.pone.0143591)

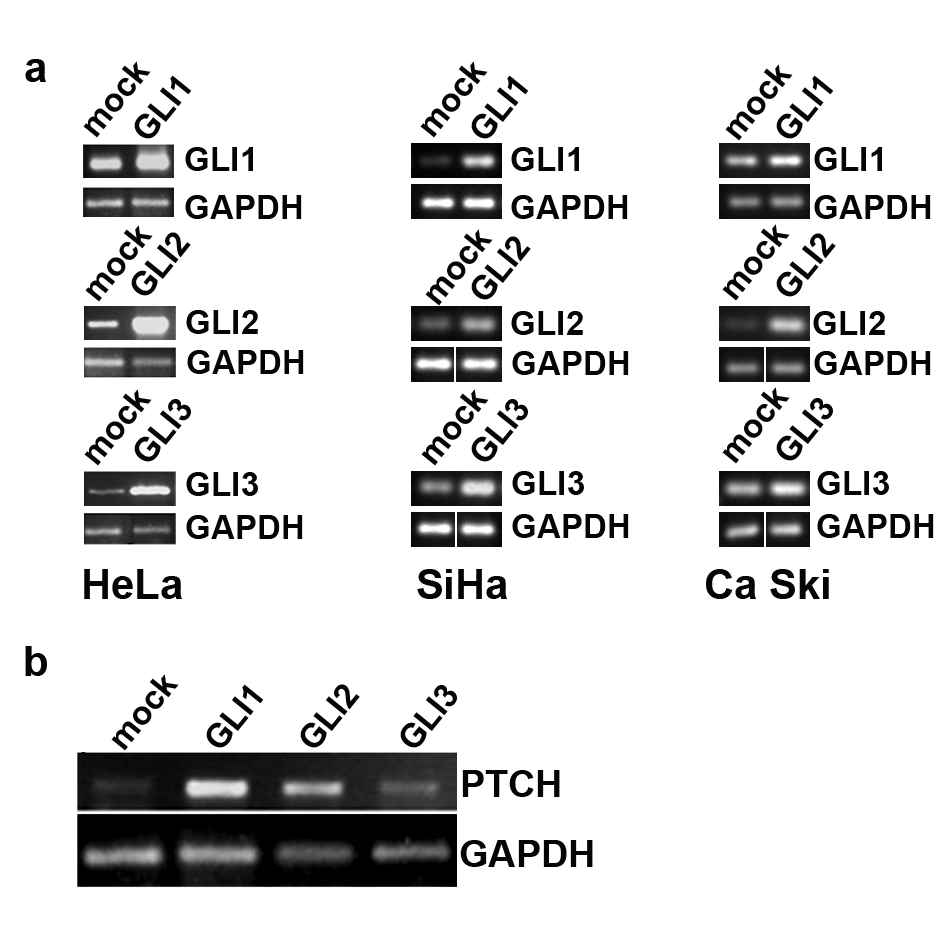

Supplement: S1 Fig — a) Overexpression of each GLI transcription factor in HeLa, SiHa and Ca Ski cells. b) The effect of GLIs overexpression on PTCH gene in HeLa cells. (TIF) [file pone.0143591.s001.tif]

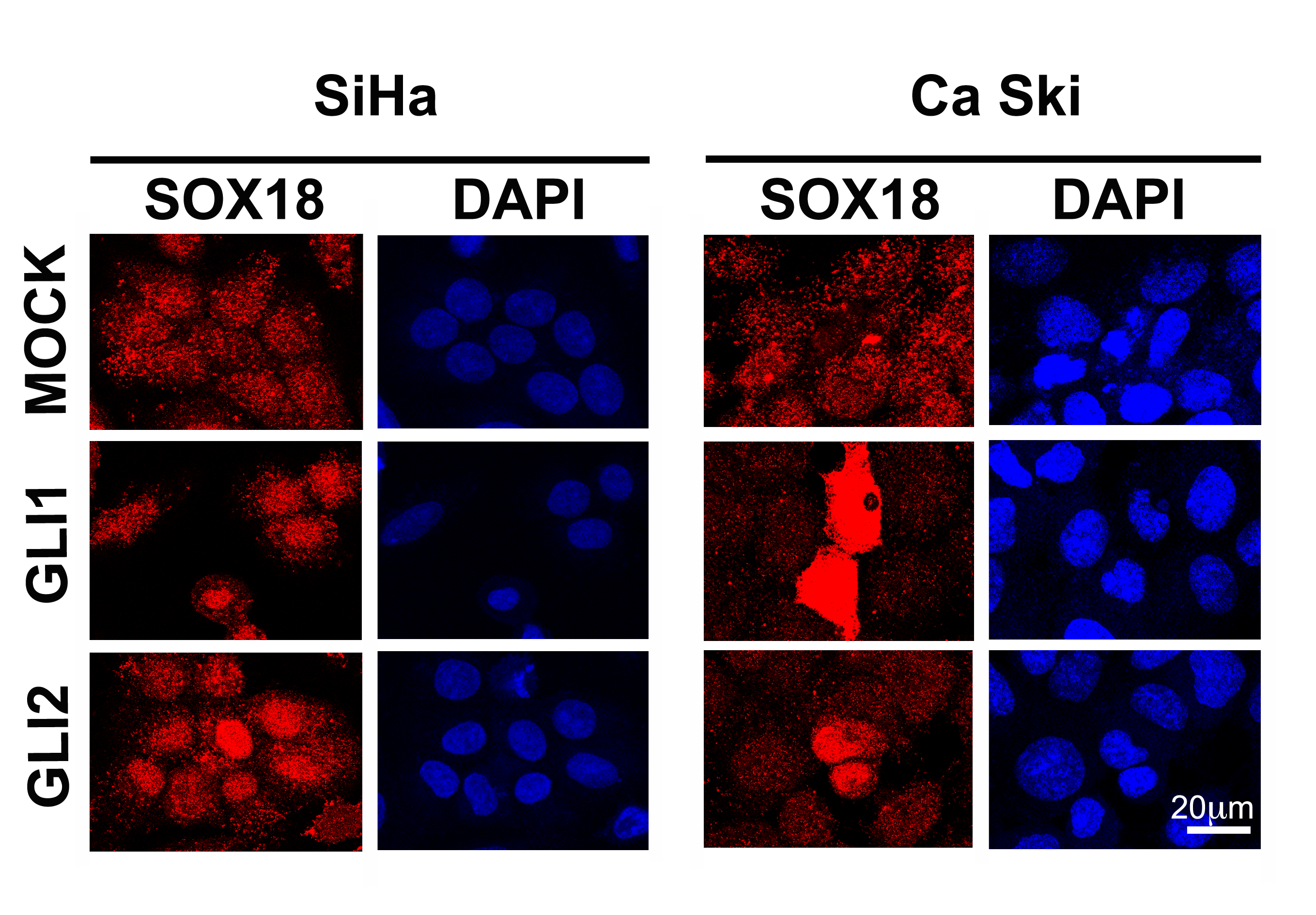

Supplement: S2 Fig — (TIF) [file pone.0143591.s002.tif]
